# Role of radiomics in predicting lymph node metastasis in gastric cancer: a systematic review

**DOI:** 10.3389/fmed.2023.1189740

**Published:** 2023-08-16

**Authors:** Francesco Miccichè, Gianluca Rizzo, Calogero Casà, Mariavittoria Leone, Giuseppe Quero, Luca Boldrini, Milutin Bulajic, Domenico Cristiano Corsi, Vincenzo Tondolo

**Affiliations:** ^1^U.O.C. di Radioterapia Oncologica, Fatebenefratelli Isola Tiberina-Gemelli Isola, Rome, Italy; ^2^U.O.C. di Chirurgia Digestiva e del Colon-Retto, Fatebenefratelli Isola Tiberina-Gemelli Isola, Rome, Italy; ^3^U.O.C. di Chirurgia Digestiva, Fondazione Policlinico Universitario A. Gemelli IRCCS, Rome, Italy; ^4^U.O.C. di Radioterapia Oncologica, Fondazione Policlinico Universitario A. Gemelli IRCCS, Rome, Italy; ^5^U.O.C. di Endoscopia Digestiva, Fatebenefratelli Isola Tiberina-Gemelli Isola, Rome, Italy; ^6^U.O.C. di Oncologia Medica, Fatebenefratelli Isola Tiberina-Gemelli Isola, Rome, Italy

**Keywords:** radiomics, gastric cancer, lymph node metastasis, predictive model, systematic review

## Abstract

**Introduction:**

Gastric cancer (GC) is an aggressive and clinically heterogeneous tumor, and better risk stratification of lymph node metastasis (LNM) could lead to personalized treatments. The role of radiomics in the prediction of nodal involvement in GC has not yet been systematically assessed. This study aims to assess the role of radiomics in the prediction of LNM in GC.

**Methods:**

A PubMed/MEDLINE systematic review was conducted to assess the role of radiomics in LNM. The inclusion criteria were as follows: i. original articles, ii. articles on radiomics, and iii. articles on LNM prediction in GC. All articles were selected and analyzed by a multidisciplinary board of two radiation oncologists and one surgeon, under the supervision of one radiation oncologist, one surgeon, and one medical oncologist.

**Results:**

A total of 171 studies were obtained using the search strategy mentioned on PubMed. After the complete selection process, a total of 20 papers were considered eligible for the analysis of the results. Radiomics methods were applied in GC to assess the LNM risk. The number of patients, imaging modalities, type of predictive models, number of radiomics features, TRIPOD classification, and performances of the models were reported.

**Conclusions:**

Radiomics seems to be a promising approach for evaluating the risk of LNM in GC. Further and larger studies are required to evaluate the clinical impact of the inclusion of radiomics in a comprehensive decision support system (DSS) for GC.

## Introduction

Gastric cancer (GC) is the third cause of cancer-related deaths in Western countries (1). Lymphadenectomy represents a main step in the multimodality management of GC, improving the oncological outcome with perioperative chemoradiotherapy (2–6). Adequate lymphadenectomy is of paramount importance to adequately stage and establish the prognosis of GC patients, which is negatively influenced by the presence of lymph node metastases (LNMs) (5, 7, 8). In fact, LNM represents an important prognostic factor that influences cancer-specific survival in patients with GC (9–14). If lymph node (LN) harvest is inadequate for stage defining, the lymph node ratio (LNR) (15) and the log odds of positive lymph nodes (LODDS) (16–18) could be used as prognostic factors for oncological outcome after curative gastrectomy, independent of the number of LNs harvested (16–21). The association between LNR and survival is stronger, particularly in cardia GC, in which the risk of cancer-specific death seems to be two to three times higher if the ratio is over 33%. Moreover, the role of LN micrometastasis (0.2–2.0 mm in size) in the pathogenesis of cancer recurrence after surgical resection was analyzed (22), and the occurrence of LN micrometastasis at the pathological examination seemed to be a negative prognostic factor strictly related to a worse oncological outcome due to cancer recurrence, especially hematogenous and peritoneal metastases (23–26).

Considering its important prognostic role and subsequent therapeutic implications in GC patients, a critical point is represented by the opportunity to accurately predict and evaluate LNM occurrence risk before making treatment decisions, which is extremely necessary (27, 28). Several tools have been suggested and evaluated to predict the presence of LNMs. Furthermore, several studies have analyzed the effectiveness of sentinel LN biopsy, but its role remains controversial due to its high false-negative rate (7–46.4%) (29–31). A valid instrument for the staging and diagnosis of loco-regional LNM is endoscopic ultrasonography with fine needle aspiration, but the accuracy of this tool is operator-dependent (32). Molecular biomarkers have also been analyzed as predictors of LNMs, but the applications of these tools are still under examination, considering their high cost and significant technological requirements (33, 34).

The extent of lymphadenectomy represents a subject of debate in GC, especially between Western and Eastern countries. In Western countries, D2 lymphadenectomy is considered mandatory for radical surgical management of GC. As reported in the randomized Dutch D1/D2-trial and Italian Gastric Cancer Study, the long-term survival of patients who underwent D2 lymphadenectomy (without routine pancreatosplenectomy) was significantly better than those who underwent D1 lymphadenectomy, both in early compared with locally advanced GC, without significant differences in terms of morbidity and mortality if performed in high-volume centers and by expert surgeons (5, 8). However, the need to perform an extent lymphadenectomy should be discussed according to tumor location, clinical staging, and related pattern of LNM, as reported in the guidelines of the Japanese Gastric Cancer Association (JGCA) for cT1N0 GC (35–39).

In oesophagogastric junction cancer, an important factor for establishing the correct surgical procedure, especially in Siewert's type II tumors, is represented by the presence of LNM in the mediastinum. In Siewert type II oesophagogastric junction cancer, the incidence of mediastinal LNM ranged between 5 and 25% (40, 41) but the accuracy of diagnostical tools (computed tomography and endoscopic ultrasonography) in establishing the presence of LNM is not yet highly acceptable (42). Having preoperatively knowledge about the presence or absence of mediastinal LNM could guide surgeons in performing better surgical procedures on patients in terms of oncological radicality than in terms of better post-operative morbidity. Several previous studies have defined the clinical indicator for predicting mediastinal LNM without obtaining significant results in terms of diagnostic accuracy (43–47).

In this context, radiomics is considered a valid tool for discovering new imaging biomarkers by converting digital radiological images into quantitative characteristics of the tumor (48–50). The potential role of radiomics in increasing the diagnostic and prognostic value of clinical-radiological features of a neoplasm has already been demonstrated in lung, prostate, brain, liver, and colorectal cancers (51). Moreover, new artificial intelligence technologies and algorithms can contribute to radiomic analysis methodologies by increasing their diagnostic power (52).

This study aims to systematically collect all available evidence on radiomics-based prediction models of LNMs in patients with gastric cancer.

## Materials and methods

### Research strategy

We followed the recommendations of the Preferred Reporting Items for Systematic Reviews and Meta-analyses (PRISMA) (53). A systematic PubMed/MEDLINE search was performed using the following search strategy: “[‘radiomic' (All Fields) OR ‘radiomics' (All Fields)] AND {‘stomach neoplasms' (MeSH Terms) OR [‘stomach' (All Fields) AND ‘neoplasms' (All Fields)] OR ‘stomach neoplasms' (All Fields) OR [‘gastric' (All Fields) AND ‘cancer' (All Fields)] OR ‘gastric cancer' (All Fields)}.” Only original articles on radiomics applications in GC characterization were selected. Papers published between 1 January 2000 and 15 February 2023 were considered for this analysis.

### Inclusion and exclusion criteria

The inclusion criteria were as follows: (1) articles on radiomics; (2) original articles; (3) papers on LNM prediction in GC. The exclusion criteria were as follows: (reason 1) articles not related to radiomics; (reason 2) not original articles (e.g., reviews, editorials, letters, congress communications or posters, and book chapters); (reason 3) articles not referring to GC in humans; (reason 4) articles not written in English, French, Spanish, or Italian; and (reason 5) articles that have not considered LNM prediction.

### Systematic review workflow

All the papers were selected and analyzed by a multidisciplinary board of two radiation oncologists (ROs; CC and ML) and one surgeon (GR), followed by independent validation by three experts in GC (one RO, FM; one surgeon, VT; and one medical oncologist, DCC). CC and ML selected the studies independently, and GR helped reach a consensus in case of discordance. All three researchers validated the final paper selection; FM, VT, and DCC were involved in the case of discordances in the final evaluation of the paper selection. All papers were screened on the bases of their title and abstract. Then, their eligibility was assessed after a full-text examination. Data extraction and synthesis were conducted by ML, CC, and GR, while FM, VT, and DCC were involved in the case of discordances. The extracted data included the first author, year of publication, number of patients (organized into training, internal, and external validation cohorts), imaging modalities, number of features, predictive model, area under the curve (AUC) if available (organized into training, internal, and external validation cohorts if available), TRIPOD classification, and Radiomics Quality Score (RQS) (48). The results are summarized in a Table 1.

**Table 1:** Quantitative synthesis of the 20 selected articles.

**First author, year**	**N°of patients (training/internal validation/external validation)**	**Imaging modality (phases or sequences)**	**N°features**	**Predictive model**	**AUC training/internal validation/external validation**	**Tripod classification (RQS, %RQS)**
Jiang et al. (54)	312/360/1017	CT (portal venous phase)	269	Nomogram	pN1 vs. pN0: 0.802 pN2 vs. pN0: 0.892 pN3 vs. pN0: 0.949	3 (15, 41.67%)
Chen et al. (55)	71/47/28	MRI (DWI sequences)	1,305	Nomogram	0.857/0.878	3 (15, 41.67%)
Feng et al. (56)	326/164	CT (venous phase)	93	Automatic clinical DSS	0.824/0.764	2a (12, 33.55%)
Yang et al. (28)	118/52	CT (arterial phase)	2,394 (1,561 on the primary tumor, 833 on the nodes)	Radiomic signatures	0.991/0.882	1b (14, 38.89%)
Dong et al. (57)	225/454/51	CT (multiphases)	1,203	Nomogram	Overall C-indexes: 0.821/0.797/0.822	3 (18, 50%)
Wang et al. (58)	197/50	CT (arterial phase)	844	Nomogram	0.886/0.881	2a (12, 33.33%)
Gao et al. (59)	486/240/42	CT (portal venous phase)	859	Nomogram	0.92/0.86	3 (15, 41.67%)
Li et al. (60)	136/68	CT (arterial and venous phases)	527 (136 deep learning features, 391 radiomics features)	Nomogram	0.84/0.82	2a (14, 38.89%)
Sun et al. (61)	531/975/112	CT (venous phase)	269	Radiomics score	0.0667/0.823	3 (23, 63.89%)
Liu et al. (62)	156/29	18F-FDG PET/CT	2,100 (1,050 PET-based features, 1,050 CT-based features)	Machine learning model	n.a./0.882	2b (15, 41.67%)
Wang et al. (63)	80/79	CT (venous phase)	546	Nomogram	0.915/0.908	2a (16, 44.44%)
Gao et al. (64)	308/155	CT (portal venous phase)	859	Nomogram	0.91/0.89	2b (14, 38.89%)
Wang et al. (65)	340/175	CT (venous phase)	352	Nomogram	0.896/0.814	3 (12, 33.33%)
Xue et al. (66)	127	18-FDG PET/CT	71	Nomogram	0.81	1b (12, 33.33%)
Zeng et al. (67)	388/167/79	CT (portal venous phase)	107	Machine learning model	0.901/0.915	3 (15, 41.67%)
Zhang et al. (68)	367/156	CT	48	Machine learning model	0.796/0.762	2a (12, 33.33%)
Xue et al. (69)	134/59/31	18F-FDG PET/CT	136 (71 PET-based features, 65 CT-based features)	Nomogram	0.861/0.897	3 (17, 47.22%)
Guan et al. (70)	242/105	CT (arterial phase)	401 (375 deep learning features, 26 radiomics features)	Nomogram	0.997/0.991	2a (15, 41.67%)
Yang et al. (71)	193/98	CT (venous phase)	98 [49 on the primary tumor region (C1), 49 on the peri-tumor region (C2)]	Radiomics score	0.779/0.724	2b (15, 41.67%)
Xue et al. (72)	127	18-FDG PET/CT	71	Radiomics Score	0.882	1b (14, 38.89%)

N°, number; CT, computed tomography; MRI, magnetic resonance imaging; 18F-FDG PET/CT, 18 F-fluorodeoxglucose positron emission tomography/computed tomography; DWI, diffusion-weighted imaging; DSS, decision support system; pN, pathological stage; n.a., not available; RQS, radiomics quality score; and RQS%, radiomics quality score in percentage.

## Results

### Characteristics of the included studies

**Figure 1:**
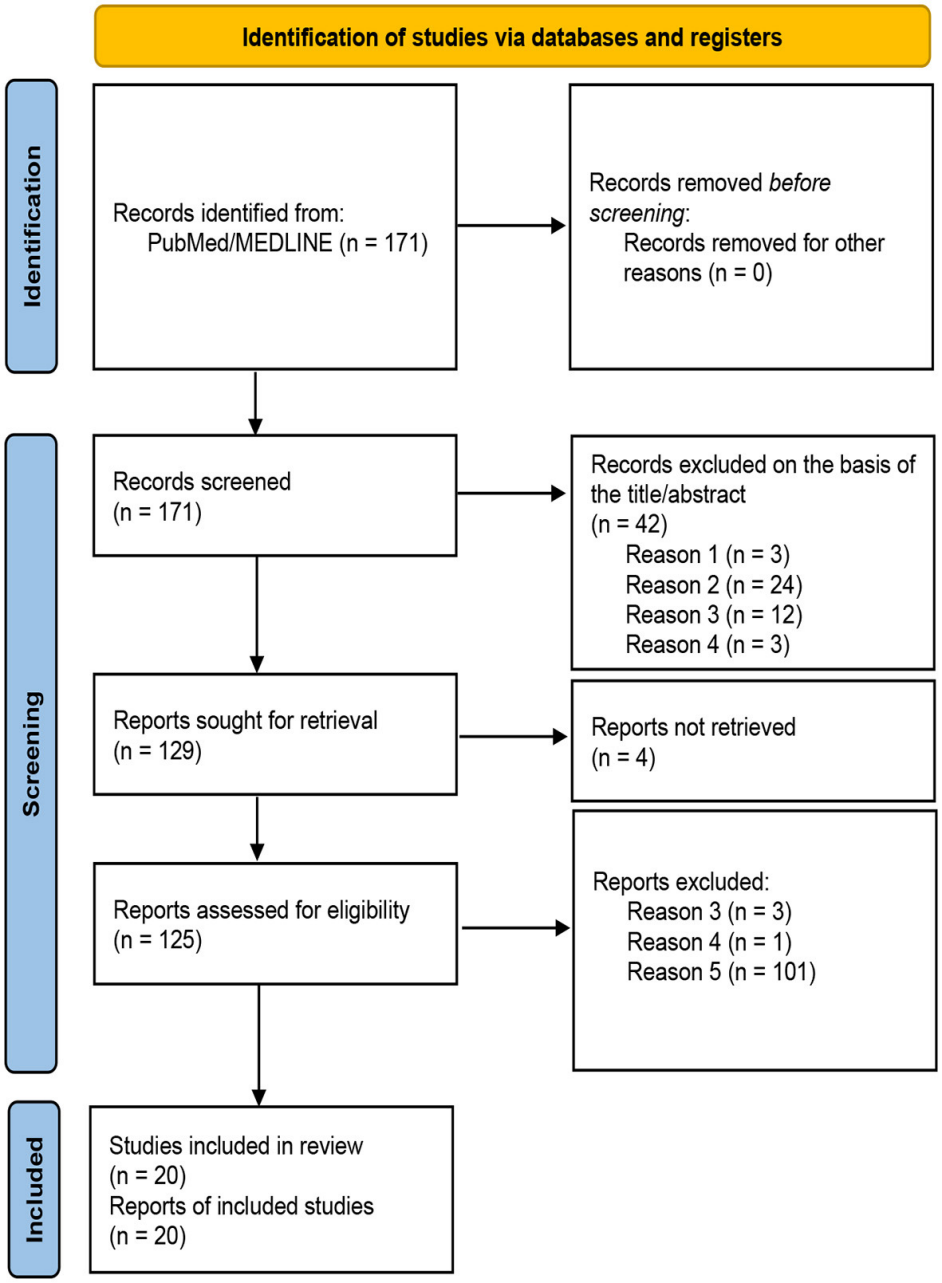
PRISMA 2020 flow diagram for new systematic reviews, which included searches of databases and registers only (53).

A total of 171 studies were obtained using the search strategy mentioned on PubMed/MEDLINE. Of these, 129 papers were selected based on the title and abstract, according to the previously described criteria. The selection process, shown in Figure 1, led to the identification of 20 eligible studies based on full-text analysis (three papers discarded according to exclusion criterion three; one paper discarded according to exclusion criterion four, and 101 papers discarded according to exclusion criterion five), and these were selected for the analysis of the results. All the studies included in the analysis are retrospective, with their year of publication ranging from 2019 to 2023. The most frequent imaging modality was CT (15 papers, 75%), followed by 18-FDG PET/CT (4 papers, 20%), and MRI (1 paper, 5%). The number of features per study ranged from 48 to 2,394.

### Narrative synthesis of the results

#### CT-based radiomics studies

Sun et al. (61) conducted a retrospective and prospective multicentre study, analyzing data from 531 patients as a training cohort, 975 patients as an external cohort, and prospectively enrolling 112 patients as a validation cohort. The study, based on preoperative CT images, developed a radiomic score (Rad-score) and correlated it with the presence of metastases in each LN station. In both the external and prospective validation cohorts, the Rad-score turned out to be the most important predictor for the detection of LNMs and remained a significant and independent factor in multivariate analysis in all patient groups. As stated by the authors, the novelty of this study was the evaluation of the risk of metastases for each LN station. This allowed for the establishment of whether neoadjuvant therapy is necessary, thereby also allowing for a tailor-made selection of the extent of surgery required and an understanding of the LN stations that should be treated.

Jiang et al. (54) also analyzed preoperative CT images to develop a radiomic signature and subsequently integrate it (by a Rad-score) into a nomogram, along with other clinical-pathological factors (degree of differentiation, Ca19.9 level, cT stage, and cN). The results showed a statistically significant correlation between the Rad-score and LN status; the higher the stage (pN0, pN1, pN2, and pN3), the higher the score. This made it possible to implement a practical tool, which, given the characteristics of the patient and the tumor, could estimate the probability of the LN stage.

Wang et al. (58) analyzed the CT images of 247 patients (197 in the training cohort and 50 in the test cohort) with pathologically confirmed gastric cancer, to define the LN stage. The nomogram consisted of radiomics scores and the CT-reported LN status and showed excellent discrimination in the training and test cohorts with AUCs of 0.886 (95% CI, 0.808 to 0.941) and 0.881 (95% CI, 0.759 to 0.956), respectively. The model outperformed routine CT in the discrimination of cases with LN metastasis, with the accuracy increased to 80–84%.

Wang et al. (65) focussed on the prediction of 10 LNMs in advanced proximal GC from a cohort of 340 patients (training cohort) and 175 patients (test cohort) from two different centers. They built a radiomics nomogram based on a multivariable analysis of radiomic signature and clinical characteristics, in particular the Rad-score and CT- No.10 LNMs' status, which were defined by the radiologists and selected as two independent predictors.

Wang et al. (63) analyzed the radiomic characteristics of both the tumor and LN station N 3 in patients with early-stage GC (T1-T2) and developed a nomogram that could predict the presence of LNMs. In view of the good results obtained, the authors applied the same method to a more limited sample for LN station 4, using it as a validation set.

Yang et al. (28) developed and validated a radiomics method based on multi-step feature selection to identify the preoperative LN status in 170 patients with GC (113 patients with positive LNs and 57 patients without metastases, surgically treated and pathologically confirmed; 118 patients for training and 52 patients for validation), by incorporating tumor and LN radiomics features. The results confirmed that taking into account the characteristics of both the primary tumor and the LNs improves the predictive capabilities of the radiomic model and that even better performances are obtained from the radiomic-clinicopathological model. Therefore, this method can be useful for guiding therapeutic choices in patients with GC (especially for the group of patients in stage T2, of a diffuse and moderately/well-differentiated type).

Yang et al. (71) developed a radiomics model combining features from the tumor and peri-tumor regions for predicting LNM and prognosis by analyzing the data of 291 patients (193 for training and 98 for validation) who had undergone preoperative contrast-enhanced abdominal CT scanning, radical gastrectomy, and extended LN dissection. The authors focussed their analysis on the peritumoural area within 5 mm, which includes many morphological changes, such as extramural venous invasion and the presence of small LNs. They concluded that the radiomic approach in this area can provide important information related to the number of metastatic LNs. However, the radiomic model proved to be unstable, probably due to the relatively limited sample size analyzed. Thus, a larger number of patients will be required to build a reliable predictive model.

The study published by Dong et al. (57) is the only international study among those analyzed that used an external validation cohort from Italy (51 patients). The authors analyzed the data of 679 locally advanced GC patients from five centers in China, divided into four cohorts: a primary cohort for training (PC, *n* = 225) and three validation cohorts (VC1, *n* = 178; VC2, *n* = 145; and VC3, *n* = 131), along with an international cohort. There was a significant positive correlation between the deep learning radiomic nomogram (DLRN) score and the pathological *N* stage. The stratification analysis showed that DLRN performance was independent of clinical factors such as age, gender, histology, tumor location, or even technical factors such as CT system version and slice thickness. The DLRN could well discriminate non-N0 groups from N0 in all cohorts. Using this model to guide lymphadenectomy (where non-N0 patients receive lymphadenectomy and N0 patients do not), the decision curves indicated that the DLRN could provide more benefits to patients than single signatures, the clinical model, the non-lymphadenectomy scheme, and the all-lymphadenectomy scheme. The importance of this study lies in the fact that DLNR was tested and showed high predictive ability and reproducibility across different centers. However, the international cohort was limited, and the authors concluded that a further prospective study conducted on a larger scale, including both Asian and non-Asian populations, could further improve the model's performance, which would benefit from the diverse sample.

Guan et al. (70) used a deep learning features model to build a nomogram. The authors selected the data of 347 patients (training cohort: 242 and test cohort: 105) to extract radiomic deep learning features. All the clinical, pathological, and laboratory data, including age, gender, tumor location, tumor morphology, albumin, neutrophils, lymphocyte, CEA level, CA742 level, CT-reported LN status, and deep learning feature scores were evaluated by performing univariate and logistic regression analyses. The results showed that the deep learning feature scores and the LN status reported by CT were independent factors. Finally, a classification model using deep learning and radiomics features together was evaluated. Surprisingly, however, the performance did not improve after the two were combined. The authors concluded that there might be a reproducibility issue with human-defined radiomics. Contrarily, deep learning, which does not need human pre-definition, is more independent, and its use may improve versatility and accuracy.

Zhang et al. (68) aimed to build a non-invasive measurement based on pre-trained deep learning models and compared their performance with that of the traditional radiomic model. The authors collected the data of 523 patients who had pathologically confirmed LAGC and randomly divided them into the training cohort (367 patients) and the testing cohort (156 patients). Three groups of hand-crafted radiomic features were analyzed: shape features, histogram statistics, and second-order features, while learning features were extracted from preoperative CT images using five pre-trained convolutional neural networks. Moreover, a support vector machine (SVM) was employed as the classifier. The authors explored the possibility of improving the model by integrating radiomics and deep learning features. Additionally, they explored the addition of clinical factors, but the prediction performance did not improve.

Zeng et al. (67) aimed to develop and validate a predictive model by combining deep transfer learning (DTL), radiomics, and clinical features for LNM. They focussed on 555 patients with early GC (EGC). They were randomly split into two cohorts: the training cohort (*n* = 388) and the internal validation cohort (*n* = 167), along with 79 patients from another center who were regarded as the external validation cohort. Clinical parameters such as age, gender, tumor size as the maximal diameter, depth of tumor infiltration, histological grade, Lauren type, ulcer, and lymphovascular invasion were considered. The best performance of the model was obtained by combining the clinical variables with the radiomic ones and with the DTL features.

Gao et al. (64) conducted a retrospective study on 726 patients, who were separated into the training cohort (*n* = 486) and the validation cohort (*n* = 240), along with 42 patients whose data were taken from The Cancer Genome Atlas (TCGA)-Stomach Adenocarcinoma (STAD) dataset and used as the external testing cohort. Patients were stratified based on the pathological N stage into LNM- (pN0) and LNM+ (pN1-3) groups. The values of each radiomics feature between the two groups were compared. The radiomics signature showed good predictive performance in the testing cohort (the TCGA-STAD dataset that enrolled non-Asian patients). A radiomics-based model, which combined CA72-4, the radiomics signature, and the CT-reported LN status, was established and demonstrated as the nomogram, but it was not possible to conduct external validation on this model because the TCGA-STAD dataset does not include information about blood biomarkers. The authors conducted a subgroup analysis in the early gastric stage (143 patients, 23 of whom were LNM+ at surgery). The radiomic signature and the radiomics-based model pattern demonstrated a good ability to discriminate LN status in this group, superior to that of CT.

In another study, the same authors (59) analyzed 463 patients (308 for the training dataset and 155 for the validation dataset) with EGC (T1a-T1b) who received radical gastrectomy with R0 resection and D2 lymphadenectomy to build a radiomics signature-based nomogram. The authors pointed out that the sensibility of CT in detecting LNM in this study was low (only 21.7%) and was outperformed by the radiomics signature. In early-stage GC, it is difficult to identify and delineate the lesions, but in this study, rather than having a precise segmentation of the lesions, the peritumoural tissue was also included in the VOI for feature extraction. In fact, the authors considered that the peritumoural microenvironment could provide useful information to more fully characterize tumor heterogeneity. It was also considered that, although many lesions of EGC cannot be detected on CT images, non-visible lesions at CT are highly suggestive of the T1a stage and rarely have metastatic LNs. The limitation of this study, as with many similar studies, lies mainly in the limited sample obtained from a single center.

Li et al. (60) analyzed the data of 204 patients randomly splatted into a training (136 patients) and a validation (68 patients) cohort. CT scans were performed using two versions of dual-energy CT. Both deep learning features (n 136) and handcrafted features (n 391) were extracted. Based on the training set, two deep convolutional neural networks (DCNNs) were constructed and trained to extract deep learning features from two groups of ROIs, and 68 arterial phase (AP) features and 51 venous phase (VP) features were analyzed to build two radiomics signatures. The radiomic nomogram proved to be useful in patients' stratification based on the risk of the presence of LNM, and it was superior to the single energy and clinical models. According to the monocentric experience, the authors concluded that a multicentric external validation is needed to assess and confirm the results.

Feng et al. (56) used a machine learning approach to develop and validate an automatic clinical decision support system (DSS) for preoperative reporting of the risk of LNM. The authors began with a dataset of 490 patients with GC who underwent primary radical gastrectomy with regional LN dissection, according to the Japanese GC treatment guidelines 2010 (version 3). The developed DSS model showed higher sensitivity and diagnostic accuracy than the conventional staging criterion (CSC). As confirmed by the authors, there is a need for external validation, preferably from a larger international cohort, to develop an automatic method of lesion segmentation that would make the process easier and faster.

#### PET/CT-based radiomics studies

Xue et al. (69) used 18F-FDG PET/CT and analyzed radiomics features in 224 GC patients from two centers. A predictive model was developed for 134 patients in the training cohort and subsequently validated in two groups, internal (59 patients) and external (31 patients). The model combined PET/CT radiographic signatures and more conventional risk factors such as Ca19.9 and PET/CT radiological diagnosis. The radiomic nomogram was found to have a higher predictive value than the PET/CT scan, and the radiomic model alone showed an improvement in sensitivity, which was detected in all three cohorts, while specificity was slightly decreased. The authors concluded that the X-ray nomogram could be a useful tool to compensate for the lack of diagnostic sensitivity of preoperative PET/CT. As the authors concluded, the main limitations of the study lie in the relatively small sample size and the fact that the results were limited to identifying only the presence of LNMs, without defining the stage and location of positive LNs.

Xue et al. (66, 72) published two more studies in 2022 and 2023, respectively. In the first study, the authors enrolled 127 patients with pathologically confirmed GC who underwent preoperative 18F-fluorodeoxyglucose (18F-FDG) PET/CT, with the aim of building and validating an 18F-FDG PET-based radiomics nomogram to predict N2-3b LNM. In the second study, the authors proposed a mixed prediction model that integrated the Rad-score and independent clinical risk factors.

Liu et al. (62) applied a machine learning process at 18F-FDG PET/CT radiomic features and developed and validated a binary model using two radiomic features to predict LNMs preoperatively. They analyzed the data of 156 patients as the training database and 29 patients as the validation cohort. The PET/CT-based radiomics model was superior to the CT-based features in discriminating LN status and could, therefore, be useful for optimizing diagnostic performance by integrating 18F-FDG PET/CT. Additionally, the authors correlated the selected features used to establish the predictive model (CT feature: Maximum 3D Diameter and PET feature: Maximum 2D DiameterSlice) with pathological characteristics traditionally associated with the process of metastasis and nerve invasion, such as vascular tumor thrombus, nerve invasion, and infiltration depth (*p* < 0.05). They concluded that the inclusion of these features could improve the performance of the radiomic model.

#### MRI-based radiomics studies

The study by Chen et al. (55) was the only study that used MRI images. The authors enrolled 146 patients from two centers (71 patients as the training cohort, 47 patients from one center as the internal validation cohort, and 28 patients from another institution as the external validation cohort). A significant correlation between LN status and radiomic nomogram was demonstrated in all three groups, and the latter obtained better results than magnetic resonance. The authors concluded that further studies with a larger sample size and with the subdivision of LN stages into more categories are needed.

A general summary of the main characteristics of the analyzed studies, including the number of patients, TRIPOD classification, RQS, and performance evaluation, is presented in Table 1.

### TRIPOD classification and RQS

According to the TRIPOD classification (73), three papers (15%) were type 1b (development and validation using resampling); six papers (30%) were type 2a (random split-sample development and validation); 15 papers were type 2b (non-random split-sample development and validation); and 8 papers (40%) were TRIPOD type 3 (development and validation using separate datasets). The RQS ranged from a minimum of 12 points (33.33%) to a maximum of 23 points (63.89%), and the median RQS was 15 (41.67%).

## Discussion

**Figure 2:**
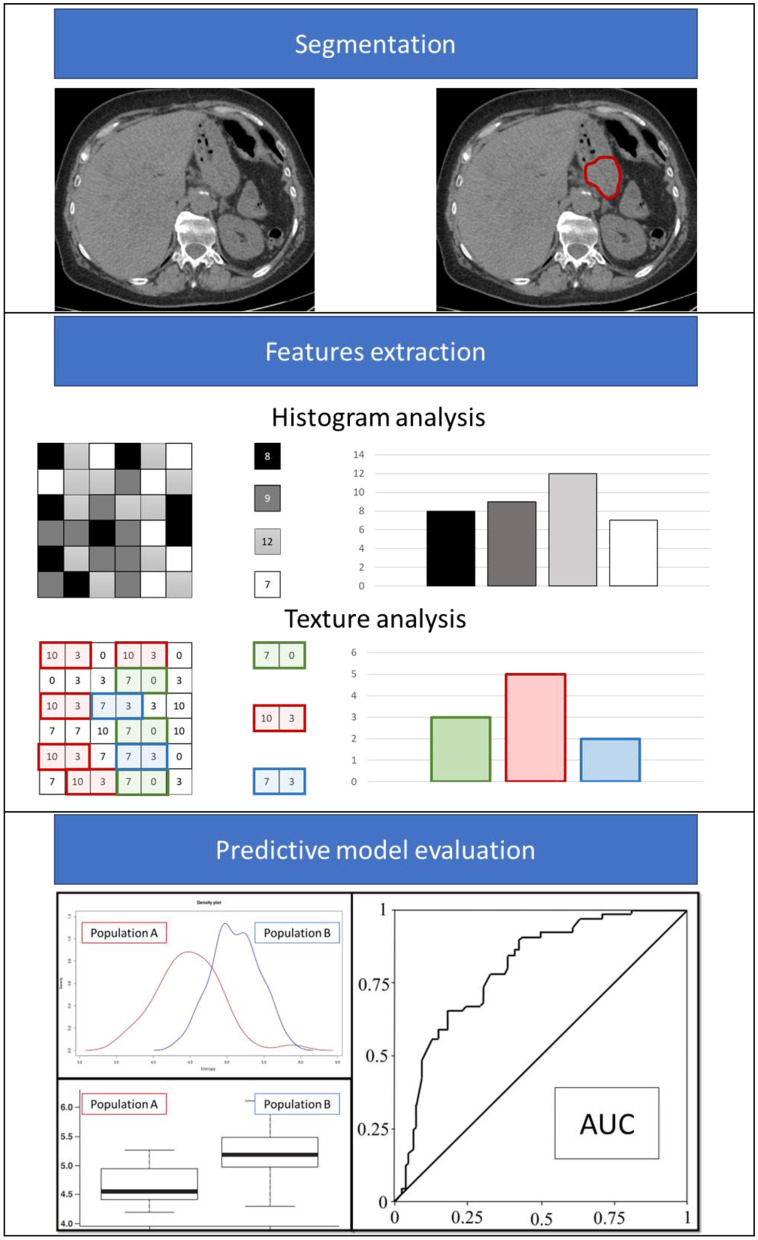
Example of the radiomics analysis workflow.

The result of this systematic review shows that radiomics is an interesting approach for evaluating LNM risk in patients with GC in various clinical settings and contexts. Figure 2 shows an example of the clinical implementation of a radiomics-based prediction model. The lack of standardization of the feature extraction process from the technical viewpoint represents one of the limitations of this methodology, which hinders the large-scale applicability of a model developed and validated in a single center. Additionally, the issue of imaging segmentation should be considered a possible second limitation: manual segmentation is burdened by operator dependence, and automatic segmentation requires dedicated and reliable software. The contextual external validation of a new model (TRIPOD 3) and/or a further external validation of an already published model (TRIPOD 4) are usually considered the gold standard for retrospective models before testing them in a prospective randomized clinical trial, especially in cases where studies are conducted with highly large cohorts of patients. In fact, the quality and reliability of the tool, especially when based on artificial intelligence, such as in machine-learning tools, is also directly related to the number of patients recruited, as well as the TRIPOD validation category.

This systematic review has several limitations. The first limitation is the use of only a single database (MEDLINE-PubMed). The second limitation is represented by the heterogeneity of the studies, which includes differences in imaging segmentation in terms of predefined target definition (primary tumor, peritumoural area, and LNs) and in terms of evaluated features, features extraction techniques, outcomes of the studies, and clinical parameters considered in the models. These factors restrict any quantitative analysis of the extracted data, making it difficult to draw clearer and synthetic conclusions. Moreover, the current stage of development of radiomics models seems to be immature, with only eight studies among the 20 selected showing a TRIPOD three classification, with low to moderate RQS levels. Finally, another limitation is that the majority of the studies recruited Asian populations, potentially limiting the high applicability of the results or at least warranting confirmation before precise application in different ethnic contexts.

In other gastrointestinal malignancies, such as rectal cancer, pancreatic cancer, and liver cancer, radiomics has been thoroughly analyzed, covering various aspects from diagnosis to treatment response assessment (74–81), the prediction of distant metastasis (82) and diagnosis (83), and even evaluation for personalization of clinical decisions based on radiomics models (84, 85). Regarding GC, only in recent years has there been a surge of papers in the scientific literature, where radiomics has been applied in different clinical settings. These settings include prognostic stratification (86–89), prediction of peritoneal lesions (90–93), assessment of response to neoadjuvant treatments (54, 94, 95), prediction of peculiar biological and molecular factors (96–100), and prediction of post-operative complications (101, 102). Within this framework, the prediction of LNM through radiomics-based tools represents one of the most explored opportunities.

In the context of an increasingly personalized and multi-omics-driven care pathway, the integration of these image analysis modalities (possibly combined with AI algorithms) within DSSs seems to be an interesting prospect, considering the clinical heterogeneity that characterizes GC patients. Radiomics represents, therefore, a promising method for the early identification of GC patients burdened with an increased risk of LNM. This scenario opens up new opportunities for selecting patients for possible intensification of locoregional treatments or remodulation of therapies already considered standard of care, based on better risk assessment.

## Conclusion

Radiomics methodology has been widely used in GC. Based on the 20 papers selected, there is evidence that it provides an interesting tool for clinicians and researchers to evaluate the risk of LNM in GC. Further and larger studies are required to incorporate radiomics parameters into a comprehensive DSS for GC and to assess the clinical benefits of improved patient risk stratification.

## Data availability statement

The raw data supporting the conclusions of this article will be made available by the authors, without undue reservation.

## Author contributions

FM and VT designed the work. GR and CC contributed to the concept of the work. GR, ML, and CC acquired and analyzed data and participated in writing the manuscript. GQ and LB acquired and analyzed data and revised the manuscript. MB, DCC, VT, and FM reviewed and revised the manuscript. All authors contributed to the article and approved the submitted version.
